# Two Cases of Cerebral Syphilis

**Published:** 1884-12

**Authors:** H. S. Kilbourne

**Affiliations:** U. S. A., Fort Shaw, Montana Territory


### Clinical Reports.

### Two Cases of Cerebral Syphilis.

By H. S. Kilbourne, M. D., U. S. A., Fort Shaw, Montana Territory.

*Case* 1. Apoplectic seizure with succeeding (left) hemiplegia; paralysis of cranial nerves (abducens, facial and hypoglossal); crossed paralysis of the facial nerve; successive relapses; partial recovery. W. D., aged 33 years, single, came under observation October 12, 1882. He was found in bed, which he was unable to leave, he stated, because he felt dizzy on attempting to rise, and had lost the use of his left arm and leg; he was ordered to the hospital, and, on examination, was found hemiplegic on the left side of the body; paralysis not complete, but nearly so; marked left-lateral deviation of the tongue; paralysis of right side of the face, nystagmus of left eye; failure of left external retina; sensation slightly blunted in the affected motor areas, but not absent; pupils apparently normal; he has not, as far as can be learned, been unconcious; patient states that he has had, for some time, a dull, heavy pain in the right lower occipital region, worse at night. On the ant. ext. aspect of left thigh, lower one-third, is an indolent ulcer, with ragged edges, irregularly oval in shape, size 2x3½ inches. On ant. aspect of both legs a number of cicatrices of a brown color; sleep disturbed; bowels constipated; appetite poor; no fever. The patient gives a history of a single hard chancre on the glans penis, contracted in December, 1879, followed by induration of the lymphatic glands in both groins, for which he had treatment lasting about two weeks, when the sore healed. Eight months subsequently (August, 1880), he had treatment for sore throat, condylomata, and for some undetermined eye trouble (iritis?). The ulcers on the legs appeared about a year after the initial lesion and have been recurrent since that time. Patient to have pot. iodidi, gram one-third, thrice daily, increased gram one, daily, until grams two, thrice daily, are taken and so continued, also unguent hydrarg. one part, vaseline nine parts, mixed, to be spread on sheet lint and applied to ulcer once daily; faradization of left side of body and right side of face, once daily, for ten minutes; a cathartic dose and nourishing diet. Under this treatment, the patient steadily improved, the ulcer rapidly healed, paralysis diminished, but did not wholly disappear, there remaining a slight paresis in all affected regions, after thirty-seven days, continuous treatment. Acne appeared on the face during the third week, when the dose of the iodide was temporarily reduced to the original quantity and pil. hydrarg., centigrams ten, ordered every night, continued until the patient was discharged from the ward, on the thirty-eighth day from admission. He was directed to continue the use of the iodide until further notice and to report himself once a week for examination. On discharge his condition was as follows: Some slowness of action of left arm and leg and of right side of face, apparent only on close inspection; general health good. After leaving the hospital he was somewhat irregular in his habits, taking alcoholics freely at intervals. On December 9th his paralysis suddenly returned, but not to the same degree as at first, and he was re-admitted to hospital for treatment. Condition as follows: Walks without support but drags left leg; left arm "numb" and not well under his control; paresis of right facial nerve; lateral deviation of tongue; diplopia, and slight strabismus convergens; fails to move the left eye outward with facility; slight pain in occiput. Treatment resumed as before, under which there was again rapid amelioration of symptoms, but recovery was less complete than at first. On January 9th he was again discharged from hospital, ordered to continue the use of the iodide, gram one, *ter in die*, and cautioned in regard to the dangers of irregularity and excesses of any sort. On February 8th was again admitted to hospital with symptoms as on second admission, but less marked, and treatment being resumed, his condition improved as before; discharged March 1st with orders as previously; re-admitted March 31st with symptoms substantially as on last admission; treatment resumed and continued until May 28th, on which date he was discharged from the military service, his condition being then as follows: Motion: Walks rather clumsily, dragging left foot slightly; muscles of right side of face under control, but not easily; grasp of left hand weaker than right; tongue deviates slightly. Sensation: There appears to be no marked difference in sensibility to points of dividers when applied to the extremities and face on either side of the body. Special sensation: Vision for both eyes $=\frac{20}{xx}$; pupils respond well to light and are symmetrical; no nystagmus; no diplopia. Hearing: Hears watch at one metre on both sides, the same watch being heard by a sharp ear at three metres. Taste slightly dulled. Cerebration: It does not appear that the patient has any marked mental disturbance. He states that his mind is clear, but, on being closely pressed, admits that his memory, as to both events and dates, is not so good as formerly; finds it more difficult to remember "tactics" than before his illness, admits that his temper is less easily excited. General condition: He is well nourished; no adenitis; no skin lesion; no pain in head or elsewhere; bowels regular; sleeps well and is cheerful.

*Case* 2. Persistent insomnia and cephalalgia, restlessness, hebetude, simulated rheumatism; not cured. W. C., aged 29 years, single. Admitted to hospital March 25, 1883. Patient, on admission, complained greatly of rheumatic pains in the extremities, especially the legs, worse at night, and of inability to sleep. Careful examination, April 5th, showed a depressed cicatrix in sulcus, behind glans penis, on supra and right-lateral aspect, and numerous nodes on internal surface of both tibiæ. None were found on cranium or elsewhere. A single enlarged lymphatic gland (post cervical) in the neck. There was some puffing about the ankles, along both flexor and extensor tendinous sheaths, where pain is also severe. Pains constant, and especially severe at night. Headaches frequent but less constant than pains. There was some slowness of thought and speech. No cutaneous lesions were observed. No sore throat. Gums a little tumid and tender (perhaps from taking potash iod., gram one, daily, since admission).

Patient states that he contracted the disease in Dec., 1874; that it was cauterized soon after, and that it was healed within a week or ten days after the latter operation; that he had an eruption of "prickly heat" (roseola?) in 1881, and that his rheumatic pains began to be troublesome in that year, and have given him more or less trouble ever since; that he has had frequent headaches, but that the pain is not localized or very severe or constant. Patient is fairly nourished; appetite fair; mind gloomy; loses much sleep, which loss he attributes to the pain in his limbs. Ordered to have potassium iodide, gram one-third, in tinct. cinch. comp., 4 c. c. *ter in die*, with chloral hydrate, grams two, in suitable vehicle, at bed-time. Progress of case: Chloral fails to procure sleep and dose increased without effect. To have tinct. opii deodoræ, 2 c. c. at bed-time, and lead and opium lotions on sheet lint, covered with oiled silk, applied over shins at night. From this he had, on the first night, three hours' sleep. He paces the floor of the ward at night, disturbing other patients. To have unguent hydrarg., dil., rubbed into each axilla and groin, once daily, alternately. Patient fails to obtain sleep from four-gram doses of tinct. opii; is extremely restless at evening and at night; exhibits much mental depression, amounting to melancholia; appetite failing; wastes in flesh; to have pot. iod. increased gradually to grams four, daily, with three centigrams morphia sulph. and one-half milligram atropia sulph., hypodermically, at bed-time.

May 20th. Patient has not improved, but obtained about four hours' sleep from injection. Mercury and iodine discontinued on account of failure of the digestion and sponginess of the gums. No salivation. To have syrupi ferri iod., ext. gentian, fluidi, āā 2 c. c., thrice daily.

May 28th. Patient not improved in respect to the pains, but general condition is better. Sleeps at night only, when injections are given, but appears to get a little sleep during the day-time. There appears to be much more restlessness and insomnia than should be due to the osteocopic pains. Throughout the succeeding months of June, July and August, the condition of this patient remained about the same as on admission. Tonic and specific treatment, including inunctions of dilute mercurial ointment over the tibiæ, and hourly baths, was continued, with occasional intermissions of the specifics until the day of his discharge from the military service, August 31, 1883, with symptoms somewhat ameliorated, but not decidedly improved.

*Remarks*—If we say that we recognize three forms of (syphilitic) cerebral disease, one in which the symptoms result from arterial occlusion, one from irritation of gummata, and one from periosteal thickening, we may fairly assume that sudden attacks of paralysis denote one, that the second has all the symptoms common to cases of tumor, and that severe pain and headache go with the last. * * * * No doubt, in some cases all three are combined, and in many, two of them.* As neither of my cases came on to the autopsy table we may place them, provisionally, in the convenient niches so sharply cut out above. A gummy deposit in the cerebral arteries, with, possibly, a syphilitic neoplasm (gumma) at the base of the brain, is the probable pathological basis of Case 1.

The extensive periosteal lesions in Case 2 would place it in the third class of Mr. Hutchinson. In regard to the symptoms, it is noted that ptosis was absent and slight strabismus convergens, due to failure of the sixth nerve, was present. The first is regarded as a common and the last as a rare symptom by many writers. The distribution of the paralysis was so peculiar, namely, that of the sixth and ninth nerves on the hemiplegic (left) side and of the seventh nerve on the right side, that doubts arise as to the accuracy of the observation; the following points are, however, distinctly recalled beyond question, namely: Left hemiplegia, left-lateral deviation of tongue, right facial paresis,

* Hutchinson, quoted by Otis, Genito-Urinary Diseases, vol. 1, p. 202. N. Y., 1883.

not involving orbicularis palpebrarum, but implicating right buccinator, nystagmus of left eye, diplopia with slight strabismus convergens. Memory fails here to support the notes of the case, that is to say, whether right or left eye turned in. Crossed paralysis of the facial nerve is not uncommon, and, according to Nothnagel*, is very characteristic of affections of the *pons* (hemiplegia alternans). Lidell reports a case† of right hemiplegia with paresis of fifth, seventh and ninth cranial nerves of the same side, and of the third nerve on the opposite (left) side. This was a case of cerebral hemorrhage, where a clot was ound in the left crus cerebri. While I have not found, in the authorities available for reference, a case more analogous than this, I am, nevertheless, inclined to trust the record of the case, for we have so great an authority as Brown-Sequard stating‡ that the disorderly grouping of nervous phenomena should lead us to interrogate syphilis as a cause. In both cases the external surface of the cranium was carefully examined for cicatrices, nodes or other manifestations of the disease, and with negative results. Loss of electric irritability of the affected nerves, and atrophy of muscles supplied by them, was not observed, but may have been present in some slight degree. If we did not know something of the natural history of these cases, that there is frequent fluctuation in the severity of symptoms and that improvement is occasionally spontaneous, the rapid recovery of case 1 might be credited to the treatment; that it was in part so due is a fair conclusion based on the rapid healing of a large, ill-conditioned ulcer.

The results of treatment in Case 2 were disappointing, and it may be fairly questioned whether the specifics were not too vigorously pushed. The mental depression was probably increased by them, and some cachexia induced. Salivation was carefully avoided with the intent of keeping the patient steadily

---

* Ziemssen Encyl. vol. xii, p. 141.

† A Treatise on Apoplexy, Lidell, N. Y., 1873, p. 147.

‡ Genito-Urinary Diseases, Van Buren and Keyes, p. 650, N. Y., 1874.

under the mixed treatment, and it does not appear that he was benefited by it. The hygienic conditions of both cases were not unfavorable ; there was plenty of fresh air, sunlight, exercise, bathing, and a regulated diet during the time of treatment in hospital.

